# Impacted Fracture through the Neck of the Humerus

**Published:** 1842-06-04

**Authors:** 


*Impacted Fracture through the anatomical Neck of the Humerus.*—Mr. Smith exhibited, at the January meeting of the Dublin Pathological Society, an example of this rare form of injury of the humerus, the only one of the kind he had ever seen, nor was he aware of such an injury having been already described. The accident occurred about eight years since, at which time the patient (a female, æt. 52) was admitted into the Richmond Hospital, under the care of the late Dr. M'Dowell, who appears to have diagnosed the injury, as it is entered in the hospital case book as "fracture of the humerus;" but it is greatly to be regretted that the appearances were not recorded. The woman was again admitted into the hospital, under the care of Mr. Adams, about five years afterwards, with an impacted fracture of the neck of the femur; one month after the occurrence of which she died of diarrhœa. The shoulder point was then examined; the arm was slightly shortened, and the shoulder not so full and round as the opposite. Upon opening the articulation, the head of the humerus was found to be sunk into the cancellated tissue of the shaft so deeply, as to be nearly on a level with the line of the anatomical neck of the bone; the great tuberosity was inclined outwards, forming a considerable curve with the outer surface of the shaft of the humerus, around the depressed head of which there was an osseous collar of newly-formed bone, largest along the inner part of its circumference.—*Dublin Journ. Med. Sci.* March, 1842.

